# The Dietary Flavonol Kaempferol Inhibits Epstein–Barr Virus Reactivation in Nasopharyngeal Carcinoma Cells

**DOI:** 10.3390/molecules27238158

**Published:** 2022-11-23

**Authors:** Chung-Chun Wu, Ting-Ying Lee, Yu-Jhen Cheng, Der-Yang Cho, Jen-Yang Chen

**Affiliations:** 1 Translational Cell Therapy Center, Department of Medical Research, China Medical University Hospital, Taichung City 40447, Taiwan; 2 National Institute of Cancer Research, National Health Research Institutes, Zhunan 35053, Taiwan; 3 National Institute of Infectious Diseases and Vaccinology, National Health Research Institutes, Zhunan 35053, Taiwan

**Keywords:** kaempferol, Epstein–Barr virus, nasopharyngeal carcinoma, dietary flavonol, kaempferol, cancer prevention

## Abstract

Kaempferol (KP, 3,4′,5,7-tetrahydroxyflavone), a dietary flavonol, has anti-cancer, antioxidant, anti-inflammatory, antimicrobial, and antimutagenic functions. However, it is unknown whether kaempferol possesses anti-Epstein–Barr virus (EBV) activity. Previously, we demonstrated that inhibition of EBV reactivation represses nasopharyngeal carcinoma (NPC) tumourigenesis, suggesting the importance of identifying EBV inhibitors. In this study, Western blotting, immunofluorescence staining, and virion detection showed that kaempferol repressed EBV lytic gene protein expression and subsequent virion production. Specifically, kaempferol was found to inhibit the promoter activities of Zta and Rta (Zp and Rp) under various conditions. A survey of the mutated Zp constructs revealed that Sp1 binding regions are critical for kaempferol inhibition. Kaempferol treatment repressed Sp1 expression and decreased the activity of the Sp1 promoter, suggesting that Sp1 expression was inhibited. In conclusion, kaempferol efficiently inhibits EBV reactivation and provides a novel choice for anti-EBV therapy and cancer prevention.

## 1. Introduction

Flavonoids are natural polyphenolic compounds that is abundant in tea, vegetables (e.g., kale, celery, cabbage, broccoli, and beans), fruits (e.g., grapes, tomatoes, strawberries, apples, and citrus), and medicinal plants (e.g., *Ginkgo* spp., *Equisetum* spp., and *Euphorbia* spp.). Kaempferol (KP, 3,4′, 5,7-tetrahydroxyflavone) is a flavonol-type flavonoid known for its antioxidant activity. Kaempferol has various biological functions, including anticancer, antioxidant, anti-inflammatory, antimicrobial, and antimutagenic activities [1,2]. It has also been found to protect the vascular, nerve, and cardiovascular systems and prevents liver fibrosis and injury [3,4]. The antiviral effect of kaempferol compared to other flavonoids, such as that of quercetin and apigenin, has not been well investigated. In the past decade, several researchers have analyzed extracts from natural plants for anti-viral activity and found that kaempferol displays potent ability against hepatitis B virus (HBV) [5,6], hepatitis C virus (HCV) [7], herpes simplex virus-1(HSV-1) [8], and poliovirus [9]. By examining kaempferol compounds, several studies have identified their broad antiviral activity against influenza virus [10], cytomegalovirus (CMV) [11], enterovirus 71 [12] and SARS-CoV [13]. Although many positive results have been documented, some studies have shown contradictory results, revealing that kaempferol may have fewer effects in blocking HSV [14], Japanese encephalitis virus (JEV) and dengue virus (DENV) [15]. Nevertheless, the antiviral activity of kaempferol warrants further investigation.

EBV is a ubiquitous virus, identified as the causative agent of infectious mononucleosis, lymphoproliferative disorders, and hairy leucoplakia. EBV is highly associated with several human malignancies including Burkitt’s lymphoma, nasopharyngeal carcinoma, and gastric carcinoma [16]. The EBV life cycle has two stages: the latent stage and lytic cycle. In the latent stage, EBV expresses several latent products, EBNAs 1–6, LMPs 1, 2A, and 2 B, and small RNAs, EBER 1 and 2. EBV initiates a lytic cascade upon stimulation by biological or chemical factors. Two immediate-early genes are expressed first, followed by a number of early and late genes, and subsequently, the viral particles are released from host cells [16,17]. EBV lytic reactivation is considered to play an important role in EBV-associated carcinogenesis. In our previous studies, we demonstrated that EBV reactivation promotes NPC carcinogenesis by inducing genomic instability [18,19,20]. If initiation of EBV reactivation was blocked, the development of NPC carcinogenesis could be significantly reduced [21,22]. Based on these results, we developed a screening platform to search for phytochemicals with anti-EBV activity. We found that sulforaphane, emodin and two flavonoids, flavones apigenin and luteolin, have the capability to inhibit EBV reactivation and subsequently block virion release [21,23,24,25]. However, whether other subgroups of flavonoids have anti-EBV activities remains unknown.

In this study, we demonstrated that the flavonol kaempferol can inhibit EBV lytic cycle initiation, which is mediated by inhibiting the transcription of EBV IE promoter activities, and eventually restrains the release of EBV virions. This study expands the application of kaempferol and may provide an alternative approach for anti-EBV therapy.

## 2. Results

### 2.1. The Cytotoxic Effects of Kaempferol in EBV-Positive NPC Cell Lines

**Figure 1:**
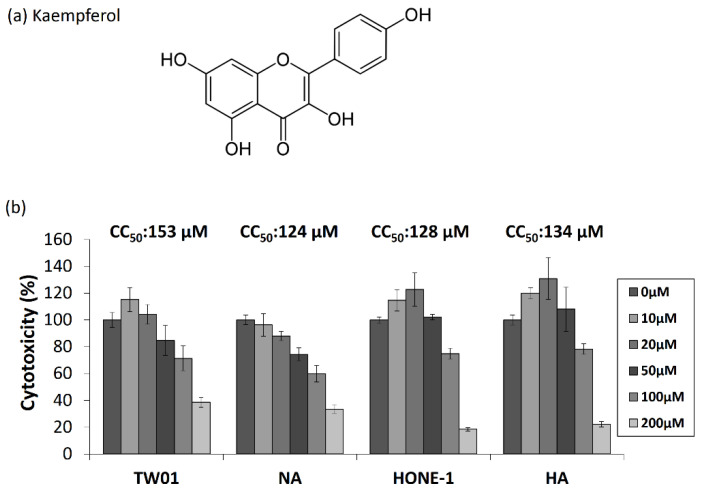
The chemical structure of kaempferol and its cytotoxicity to NPC cell lines. (**a**) The chemical structure of kaempferol. (**b**) Two sets of EBV-positive NPC cell lines and their parental counterparts (TW01/NA and HONE1/HA) are treated with various concentrations of kaempferol for 48 h. The cell viability was detected and CC_50_ values were calculated (top of each panel). The values of each sample were derived from three independent trials.

The chemical structure of kaempferol contains a polyphenol backbone with four OH groups (3,5,7-trihydroxy-2-(4-hydroxyphenyl)-4H-1-benzopyran-4-one) (Figure 1a). Before determining whether kaempferol has potential anti-EBV activity, we evaluated the susceptibility to kaempferol treatment in two pairs of NPC cell lines, EBV-positive NA and HA cells, and their parental TW01 and HONE-1 cells. NPC cell lines were seeded for 24 h. The cells were then treated with various concentrations of kaempferol for a further 24 h, and cell viability was determined using WST-1 assay. As shown in Figure 1b, kaempferol had different cytotoxic effects in the four NPC cell lines. The cytotoxicity of kaempferol in EBV-positive cell lines showed no obvious difference compared with their parental counterparts (Figure 1b), which could rule out the influence of EBV on the cytotoxic effect of kaempferol. To analyze the data more precisely, the 50% cytotoxic concentration (CC_50_) values of each cell were calculated. The CC_50_ values of kaempferol for TW01 and NA were 153 and 124 μM, and the corresponding values for HONE-1 and HA were 128 and 134 μM. To compare the cytotoxicity of kaempferol in other types of cell lines, NPC cell lines are less susceptible to kaempferol treatment [26]. Based on these results, 1–50 μM kaempferol was used for further experiments.

### 2.2. Kaempferol Treatment Inhibited EBV Lytic Protein Expression in NPC Cell Lines

**Figure 2:**
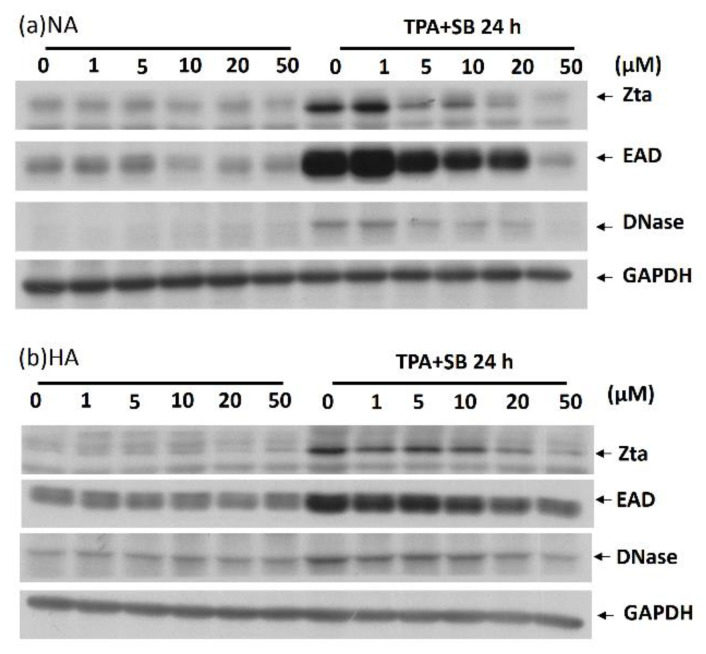
Kaempferol treatment inhibits EBV reactivation in NPC cell lines. (**a**) NA and (**b**) HA cell lines were prepared for the detection of the effects of kaempferol treatment on EBV reactivation. For detecting the enhancement of EBV reactivation, the cells were treated with kaempferol alone for 25 h (**left** panels). For detecting the inhibition of EBV reactivation, the cells were pre-treated with kaempferol for 1 h and then treated with TPA + SB for a further 24 h. All samples were collected for Western blotting analysis with antibodies against EBV lytic proteins Zta, EAD and DNase, and GAPDH was served as a loading control.

At first, we sought to determine whether kaempferol could induce EBV reactivation. The EBV-positive NPC cell lines, NA and HA, were plated for 24 h and then treated with 1–50 μM kaempferol. After induction for 24 h, cells were collected to determine the protein expression of EBV lytic genes. The immediate-early genes of EBV Zta (BZLF1) and the early genes EAD (BMLF1) and DNase (BGLF5) were detected using Western blotting. Kaempferol treatment did not induce significant expression of EBV lytic genes in NA (Figure 2a, left panel) or HA (Figure 2b, left panel), indicating that it cannot activate EBV entering the lytic cycle alone.

Next, we determined whether kaempferol inhibited EBV reactivation. For this experiment, TPA plus SB was used for EBV induction because it is an effective approach for reactivation of EBV in NPC cells within 24 h. In addition, to obtain a better efficiency of kaempferol inhibition, various amounts of kaempferol were pre-treated with plated NPC cells for 1 h prior to TPA plus SB treatment. After 24 h of induction, the cells were harvested for detection of Zta, EAD, and DNase protein expression. As shown in the right part of Figure 2a, TPA plus SB induced significant expression of EBV lytic genes in NA cells; however, following with adding kaemoferol, the expressions of lytic genes were decreased in a dose-dependent manner, strongly suggesting that kaempferol can block EBV reactivation. Similar results were also observed in HA cell lines (Figure 2b, right panel).

### 2.3. Kaempferol Treatment Decreased EAD-Expressing NPC Cells in Immunofluorescence (IFA) Analysis

**Figure 3:**
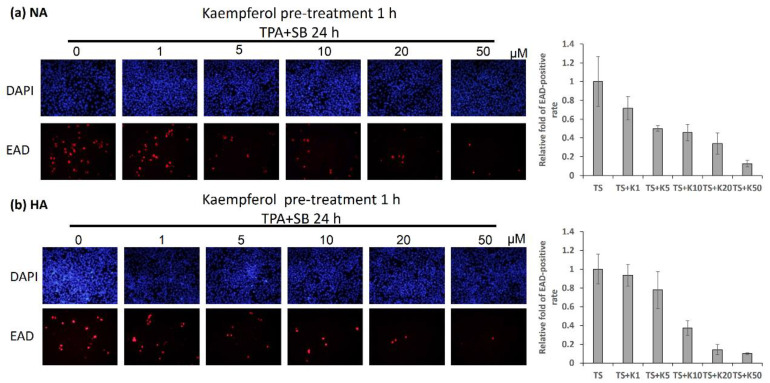
Kaempferol treatment decreased the number of EAD-positive cells. (**a**) NA and (**b**) HA cell lines were prepared for immunofluorescence analysis. To determine the inhibition of EBV reactivation by kaempferol, the cells were pre-treated for 1 h. TPA and SB were added to induce EBV reactivation. After 24 h of treatment, the cells were treated with EAD antibody and analyzed by fluorescence microscopy. The statistical results are shown as relative folds in the left panels of the figures. The values of each sample were derived from three independent trials. TS: TPA + SB; K1, K5, K10, K20 and K50: 1, 5, 10, 20 and 50 μM kaempferol.

To demonstrate whether kaempferol has anti-EBV activity, changes in cell numbers in EAD-expressing cells were calculated using IFA analysis when cells were treated with kaempferol under TPA + SB induction. As shown in Figure 3a,b, the number of EAD-positive cells gradually decreased in a dose-dependent manner in NA and HA cell lines. To analyze the decreasing tendency more precisely, quantification of EBV-positive cells was also performed. Kaempferol decreased the population of EAD-positive cells by half at a dose of 10 μM and repressed them almost completely at 50 μM (Figure 3a,b). These IFA results support the hypothesis that kaempferol inhibits EBV reactivation.

### 2.4. Kaempferol Treatment Inhibited EBV Reactivation in a Time-Course Analysis

**Figure 4:**
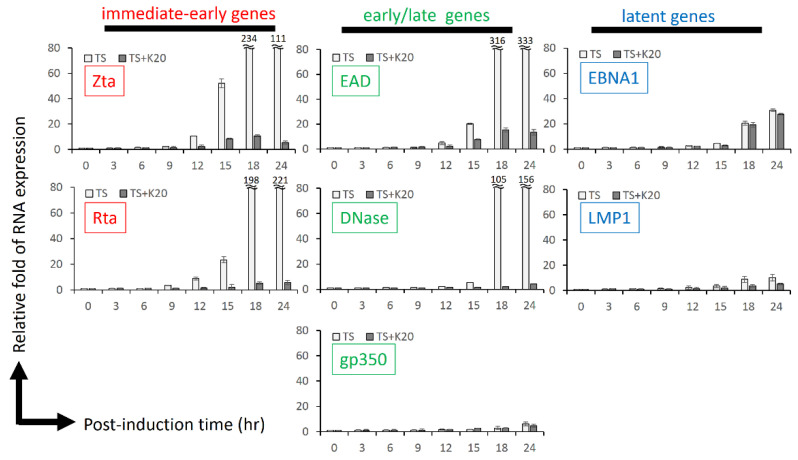
The mRNA expression of EBV lytic genes under kaempferol treatment in NPC cell line. To detect the mRNA expression of activating EBV after kaempferol treatment, NA cell lines were pre-treated with kaempferol or not, and then TPA + SB was added to induce EBV reactivation. The mRNAs extracted from each sample at different time points were subjected to RT-qPCR, as described in the Materials and Methods section. Several lytic genes (Zta, Rta, EAD, DNase, and gp350) and latent genes (EBNA1 and LMP1) were examined. The values of each sample were derived from three independent trials. The relative fold of RNA expression was represented as the relative fold of mRNA expression compared to 0 h group. TS: TPA + SB; K2: 20 μM kaempferol.

To demonstrate this phenomenon further, we designed a time-course analysis to monitor the reduction in lytic gene expression caused by kaempferol. NA cells were seeded in 6-well plates for 24 h. For the control group, TPA plus SB was added to induce EBV infection for 24 h. For the kaempferol-treated group, cells were harvested at the indicated time points for RT-qPCR analysis. Several EBV lytic genes, Zta, Rta, EAD, DNase, and gp350, were detected for their RNA expression. As shown in Figure 4, the immediate-early genes Zta and Rta were first expressed 9–12 h after TPA plus SB treatment, followed by EAD and DNase expression at 12–15 h post-induction. EBV late gene gp350 remained almost silent during the first 24 h of EBV reactivation, which is consistent with general knowledge about the EBV life cycle. In the group treated with kaempferol, all EBV lytic genes were significantly shut down, revealing that kaempferol is an effective inhibitor of EBV activation (Figure 4). Interestingly, for latent gene expression, EBNA1 and LMP1 expression increased concomitantly during EBV reactivation, similar to our previous studies. However, kaempferol treatment was less effective against EBNA1 and LMP1 (Figure 4). These results suggest that kaempferol represses the EBV lytic cycle during the initial stage.

### 2.5. Kaempferol Treatment Decreased Viral Production of EBV

**Figure 5:**
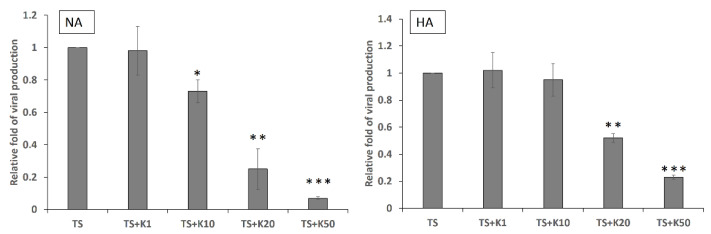
Kaempferol treatment inhibits EBV viral production in NPC cell lines. NA and HA cell lines were pre-treated with kaempferol and induced with TPA + SB for 48 h. The supernatant of each sample was treated with DNase I to remove free DNA from the virus and was subsequently treated with proteinase K to digest viral capsids. The resultant samples were subjected to qPCR analysis to detect the EBV BALF5 fragment. The values of each sample were derived from three independent trials. The relative fold of viral production is presented as a companion with the value of each sample relative to that of the TS group. (* *p* < 0.05, ** *p* < 0.01, *** *p* < 0.001) TS: TPA + SB; K1, K10, K20 and K50: 1, 10, 20 and 50 μM kaempferol.

In the above experiments, kaempferol displayed the ability to repress the RNA and protein expression of EBV lytic genes. Whether kaempferol repressed EBV virion release in the late stages remained unknown. NA and HA cells were prepared and treated with TPA plus SB for 48 h with or without kaempferol pre-treatment. The culture supernatants were collected to detect EBV DNA. The results showed that EBV DNA levels decreased with kaempferol treatment in a dose-dependent manner (Figure 5, left and right panels). Taken together, kaempferol clearly repressed EBV lytic gene expression and virion production, indicating its anti-EBV activity.

### 2.6. Kaempferol Repressed Promoter Activities of EBV Immediate-Early Genes

**Figure 6:**
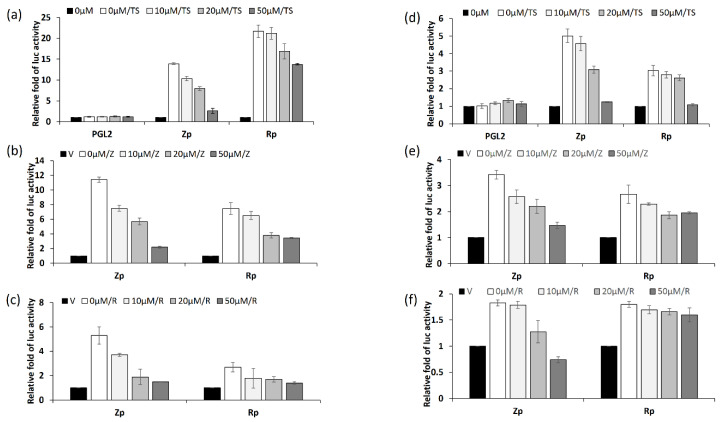
Kaempferol treatment inhibits Zp and Rp activities in NPC cell lines. The effects of kaempferol on Zp and Rp were determined using luciferase assay. NA (**a**–**c**) and TW01 (**d**–**f**) cells were transfected with Zp or Rp reporters for 3 h, followed by kaempferol pre-treatment and TPA + SB induction for a further 24 h. Cell lysates were collected to measure luciferase activity of each sample. The values of each sample were derived from three independent trials. The relative fold of luc activity was represented as the relative fold of luciferase activity compared to mock control. TS: TPA + SB; V: vector control; Z: Zta-expressing plasmid; R:Rta-expressing plasmid.

From the results described above, kaempferol treatment repressed the RNA expression of EBV lytic genes, implying that EBV inhibition by kaempferol may result from transcriptional regulation. In addition, when entering the lytic cycle, EBV first expresses Zta and Rta proteins. To address this finding, luciferase reporter assays were performed to determine the inhibitory effect of kaempferol on the Zta and Rta promoters (Zp and Rp). NA and TW01 cells were transfected with reporter plasmids containing Zp and Rp. The results showed that kaempferol inhibited Zp and Rp activities in a dose-dependent manner under TPA + SB treatment in both NA (Figure 6a) and TW01 cells (Figure 6d). In addition to chemical stimulation, we activated Zp and Rp through Zta and Rta expression. Zta expression triggered lower Zp and Rp activities than TPA + SB stimulation (Figure 6b,e), while Rta induced minimum promoter activity in NA and TW01 cells (Figure 6c,f). However, regardless of Zta or Rta stimulation, increasing the concentration of kaempferol repressed Zp and Rp activities in NA and TW01 cells (Figure 6b–f). Interestingly, kaempferol was more efficient in inhibiting Zp in both NPC cell lines (Figure 6). The results of the luciferase reporter assay indicated that kaempferol downregulated the activation of Zp and Rp during EBV reactivation.

### 2.7. Kaempferol Inhibited Zta Promoter Activity through Repressing Sp1 Expression

**Figure 7:**
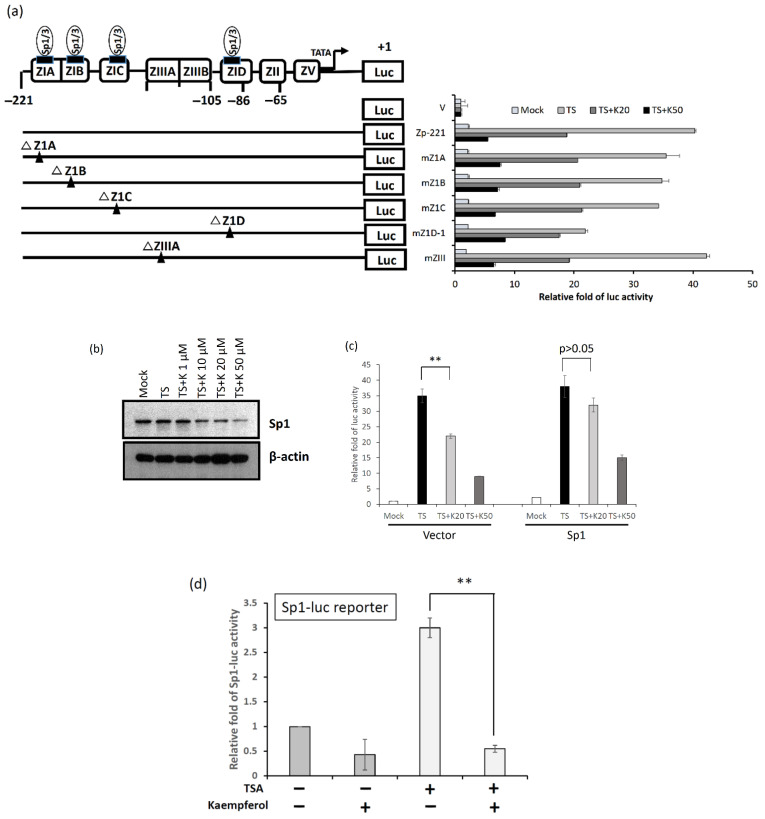
Sp1 binding domains on Zp are important for kaempferol inhibition in NPC cell lines. (**a**) NA cells treated with TPA + SB and kaempferol were transfected with the wild type Zp and its mutants harboring putative Sp1 binding sites located on Z1A, Z1B, Z1C and Z1D domains. The corresponding mutation sites were shown as a black triangle (▲) in the left diagram. The mutant with ZIIIA mutations was a positive control. (**b**) NA cells were treated with kaempferol and TPA + SB for 24 h. The cell lysates were detected for Sp1 expression. The β-actin expression was the loading control. (**c**) NA cells were firstly co-transfected with vector or Sp1-expressing plasmid and Zp reporter for 3 h, respectively. After transfection, the cells were pretreated with kaempferol and TPA + SB. After 24 h treatment, the luciferase activities of Zp were detected. (**d**) The luciferase reporter with Sp1 promoter was transfected into TW01 cells under indicated conditions: mock, kaempferol, TSA, or TSA + kaempferol treatments for 24 h. The cell lysate was detected for luciferase activities. The values of each sample were derived from three independent trials. (** *p* < 0.01) The relative fold of luc or Sp1-luc activity was represented as the relative fold of luciferase activity compared to mock control. TS:TPA + SB; K20 and K50: 20 and 50 μM kaempferol.

Sp1 has been identified as a critical factor that triggers EBV reactivation. In addition, Zta is the first protein expressed during EBV lytic initiation. Several Sp1 binding sites were identified within the Z1A, Z1B, Z1C, and Z1D domains of Zp, as shown in Figure 7a. A panel of reporter plasmids containing 3-bp mutations in the aforementioned domains was constructed to detect luciferase activity after kaempferol treatment in NA cells. The results showed that for the wild-type reporter Zp-221, kaempferol suppressed chemical-induced luciferase activity in a dose-dependent manner (Figure 7a). Moreover, the similar inhibitory tendency on kaempferol were shown for mZ1A, mZ1B, mZ1C and mZIII, but not for mZ1D-1, which the inhibitory effect was almost compensated in the treatment group of TPA + SB and 20 μM kaempferol (TS + K20) (Figure 7a, bottom panel).

We further determined whether kaempferol regulated Sp1 expression in NPC cells. TPA + SB-treated NA cells were detected for their Sp1 expression under kaempferol administration. As shown in Figure 7b, Sp1 expression was downregulated by kaempferol treatment in TPA + SB-treated NA cells. We further transfected the Sp1-expressing plasmid to resupply the SP1 protein level under the kaempferol treatment to determine whether the Zp activity can be compensated. The results displayed that activated Zp activity was repressed by kaempferol significantly; however, this repression was compensated by resupplying with Sp1 (Figure 7c). Moreover, kaempferol displayed the ability to repress Sp1 promoter activity (Figure 7d). Taken together, the results presented here suggest that kaempferol suppresses the Zp promoter by downregulating Sp1 expression.

## 3. Discussion

Recently, because of the COVID-19 pandemic worldwide, the concept of “phytotherapy” has become an attractive strategy for anti-viral therapy. Several types of flavonoids have been identified for their anti-viral bioactivities [27]. Flavonols are a subfamily of flavonoids that includes quercetin, kaempferol, and galanin. Quercetin is the most popular candidate for antiviral therapies [28]. Compared to quercetin, kaempferol has been reported less frequently for the treatment of viral infections. Kaempferol has been shown to have inhibitory activity against many types of viruses; however, its anti-EBV activity remains unknown. In this study, kaempferol showed its ability not only to inhibit EBV lytic protein and RNA expression (Figure 2, Figure 3 and Figure 4), but also eventually to release viral particles (Figure 5). To explore this underlying mechanism, kaempferol was further identified to repress EBV IE promoters by attenuating Sp1 protein levels to block EBV lytic initiation (Figure 6 and Figure 7). These results clearly demonstrate that kaempferol inhibits EBV reactivation.

On the other hand, quercetin, having a similar chemical structure with kaempferol except without a hydroxyl group at position 3′ of B ring [29], has been extensively studied for its broad-range antiviral activity. Anti-HCV activity directly inhibits HCV activity by interfering with NS2, NS3, and NS5A functions and reduces HCV-induced ROS and RNS formation during viral replication [30]. For anti-influenza virus activity, it binds to neuraminidase or RNA polymerase to block infection and viral production [31]. For anti-HIV activity, it was shown to block HIV replication against HIV integrase and topoisomerase II [31]. For fighting against HSV, HCMV and EBV, quercetin suppresses lytic gene expressions and blocks infection [32,33,34]. These results indicate that flavonols are promising candidates as antiviral agents. Following the outbreak of the COVID-19 epidemic, many flavonoids have been reconsidered for their antiviral activities against SARS-CoV-2, including flavonols. Through in silico analysis, kaempferol and quercetin have been suggested as candidates against SARS-CoV-2 by interacting with the 3CLpro, PLpro, or S protein of SARS-CoV-2 [35,36,37]. Although abundant computational data suggest that flavonols possess potent anti-SARS-CoV-2 capabilities, their actual antiviral activity still needs to be validated to complement their inhibition against SARS-CoV-2.

EBV reactivation is a critical factor in the tumourigenesis of EBV-related malignancies. Moreover, blocking EBV reactivation can efficiently reduce tumor growth. EBV lytic reactivation-based therapy is a prevalent treatment for EBV-associated malignancies [22,38]. Two opposing strategies have been applied: one is to induce lytic reactivation of tumor-resident EBV to cytolytic tumor cells [39], and the other is to prohibit EBV reactivation to relieve cancer progression [40]. Compared with the former strategy, the latter has the following advantages: (1) inhibition of the EBV lytic cycle decreases the opportunity for EBV reactivation-induced tumorigenesis, (2) blocking EBV lytic production avoids virion transmission to other cells, (3) suppressing EBV reactivation reduces the secretion of oncogenic endocrine factors from EBV-positive tumor cells, and (4) Decreasing lytic protein expression can attenuate the immune evasion of lytic EBV. Therefore, many anti-EBV agents have been explored for the inhibition of EBV lytic reactivation [40]. To date, three major categories of anti-EBV inhibitors, classified by their mechanisms, have been documented. The first category is anti-replication agents: several nucleoside or nucleotide analogues, including acyclovir (ACV), gancyclovir (GCV), cidofovir, and omaciclovir, have been shown to exert anti-EBV replication activity by causing DNA chain termination. The L(-)nucleoside analogue L(-)FMAU (clevudine) and its derivatives also show significant anti-EBV replication activity [41]. Anti-HIV drugs such as zidovudine (AZT) are also potent inhibitors of EBV replication [42] which are phosphorylated by EBV thymidine kinase [43]. Moreover, the benzimidazole derivative maribavir possesses dual anti-EBV functions: termination of viral replication and inhibition of viral kinase activity [44]. The second category includes anti-reactivation agents. Some chemical compounds (e.g., valpromide and artesunate) have been shown to have an inhibitory effect on EBV IE protein expression [45,46]. Vitamin supplements, including vitamins C and D, have been reported to be potent EBV reactivation inhibitors [47,48]. In addition, a large number of dietary phytochemicals have been identified with anti-EBV activity. Resveratrol and Epigallocatechin-3-gallate (EGCG) repress the EBV lytic cycle by blocking multiple signals [49,50]. Curcumin, sulforaphane, and emodin show inhibitory effects on EBV reactivation by inhibiting IE expression [21,23,51]. Moreover, several flavonoids have been shown to inhibit EBV reactivation. Two flavones, apigenin and luteolin, prevent IE expression and repress EBV reactivation. Interestingly, in this study, we identified that the kaempferol modulated Sp1 expression to repress EBV reactivation in the NPC cell lines. In addition, our results showed kaempferol also inhibited EBV reactivation in B cell line P3HR1 (Supplementary Figure S1). Sp1 has been demonstrated to be important for the activation of different viruses, including herpesviruses [52,53,54], HBV [55] and HIV [56], which may account for the broad spectrum of anti-viral applications of kaempferol. In fact, kaempferol also showed anti-HSV, anti-CMV, and anti-KSHV activities in our preliminary results (data not shown). This subject is worth studying further.

For exploring the underlying mechanism of kaempferol inhibition, we have provided the evidence to suggest that kaempferol repressed Sp1 expression to inhibit EBV reactivation. Another interesting question of whether kaempferol could affect nuclear translocation or the promoter-binding ability of Sp1 to inhibit EBV reactivation has been raised. In our previous study, we identified that another flavonoid, luteolin, could repress Sp1 binding to Zp and Rp [21]. Other studies, Li et al. found kaempferol reduced Sp1 expression in the nucleus [57]. Zhou et al. demonstrated that kaempferol treatment repressed Sp1 expression in both the nucleus and cytosol [58]. Based on these clues, we could reasonably speculate that kaempferol can disturb Sp1 binding but may not affect the translocation of Sp1. Except for the intercept Sp1 expression, there are two mechanisms which should be considered as candidates for this phenomenon. First, kaempferol may abolish Zta expression by interfering with c-Jun or NF-kB signaling [59]. These two signaling pathways are known to be important for Zta and Rta expression, and they have the ability to reduce NF-kB and AP-1 transcriptional activities, suggesting that this mechanism is involved in kaempferol-induced EBV inhibition. Another possible mechanism involves ROS inhibition, which prevents IE expression. ROS production is known to trigger EBV activation [60], and kaempferol is a well-documented ROS scavenger [2]. Theoretically, kaempferol can inhibit EBV reactivation by sweeping ROS levels. Nevertheless, the question of how kaempferol abolishes IE expression still needs to be explored further.

Taken together, kaempferol has many advantages as a common dietary ingredient, such as being low-toxic, low-cost, and easy to obtain, which is quite suitable for treatment of EBV-associated diseases. This study suggests that kaempferol has efficient anti-EBV activity with low cytotoxicity, and provides a novel application for kaempferol. Furthermore, we hope that this study will provide an alternative choice for anti-EBV prevention and therapy.

## 4. Materials and Methods

### 4.1. Cell Lines

TW01 [61] and HONE-1 [62] cells are EBV-negative NPC cell lines. NA and HA are EBV-positive NPC cell lines derived from EBV infecting TW01 and HONE1 cells, respectively. All cell lines were incubated in Dulbecco’s modified Eagle’s medium (DMEM) containing 10% fetal calf serum.

### 4.2. Chemicals and Antibodies

Kaempferol, 12-O-tetradecanoyl-phorbol-1,3-acetate (TPA) and sodium butyrate (SB) were purchased from Sigma-Aldrich Co. EBV antibodies anti-Zta 4F10 [63], anti-BMRF1 (EAD) 88A9 [63], and anti-DNase 311H [64] were produced in our laboratory. The anti-β-actin and anti-GAPDH antibodies were purchased from Sigma-Aldrich Co., St. Louis, MO, USA).

### 4.3. WST-1 Assay

To evaluate the cytotoxicity of kaempferol to NPC cell lines, WST-1 assay was performed according to the manufacturer’s instructions (Invitrogen, Carlsbad, CA, USA). Briefly, cells were plated in 96-well plate for 24 h. Then, the cells were incubated with various concentrations of kaempferol (0, 10, 20, 50, 100 and 200 μM) for 48 h. The cytotoxicity of each sample was detected by WST-1 assay. The absorbance was measured using microplate reader (Infinite M200, Tecan, Männedorf, Switzerland).

The standard deviation of each sample was calculated from three independent experiments in duplicate.

### 4.4. EBV Induction and Kaempferol Treatment

To address whether kaempferol induces EBV reactivation, two EBV-positive NPC cell lines, NA and HA, were plated 24 h prior to the addition of various concentrations of kaempferol (0, 1, 5, 10, 20 and 50 μM). After treatment for 24 h, the cell lysates were collected for further analysis. To determine whether kaempferol treatment inhibited EBV reactivation, NPC cell lines were plated for 24 h. Then, the cells were pretreated with kaempferol (0, 1, 5, 10, 20 and 50 μM) for 1 h, and TPA (40 ng/mL) plus SB (3 mM) were added for EBV activation. After 24 or 48 h of treatment, cells were collected for further analysis.

### 4.5. Western Blotting

The collected cell extracts were loaded onto a 10% SDS-PAGE gel and then transferred to a Hybond-C nylon membrane (Amersham Biosciences Ltd., Buckinghamshire, UK.). The blots were treated with blocking buffer (10 mM Tris-HCl [pH 8.0], 0.9% NaCl, and 4% skim milk) for 1 h, followed by incubation with antibodies. After incubation overnight, the blots were washed with washing buffer (10 mM Tris-HCl, pH 8.0, 0.9% NaCl) and treated with horseradish peroxidase-labelled goat anti-mouse IgG (1:20,000) (Amersham Biosciences Ltd., Buckinghamshire, UK). for 1 h at room temperature. After washing three times, the blots were developed with freshly prepared substrate (Amersham Biosciences Ltd., Buckinghamshire, UK), and luminescence was detected by exposure to X-ray film (Fuji).

### 4.6. Luciferase Reporter Assay

All reporters were transfected using Lipofectamine 2000 (Invitrogen, Carlsbad, CA, USA) according to the manufacturer’s instructions. Briefly, cells were prepared (2 × 10^5^/well) for Zp and Rp transfection. The Zp or Rp reporters were mixed with Lipofectamine 2000 (Invitrogen, Carlsbad, CA, USA) in Opti-MEM (Invitrogen, Carlsbad, CA, USA) for 20 min and then added to the cells. After 3 h of transfection, kaempferol (0, 10, 20 and 50 μM) was pre-treated or not for 1 h, and then TPA (40 ng/mL) and SB (3 mM) were added to induce EBV reactivation. After a further 24 h incubation, the cells were collected and lysed in HEPES buffer (0.1M HEPES, pH 7.8, 1% Triton X-100, 1 mM CaCl_2_, and 1 mM MgCl_2_). Equal amounts of the lysates and luciferase assay reagent II (Promega, Madison, WI, USA) were co-incubated for 10 min. The luciferase activity was measured using a luminescence counter (Packard). Each sample was quantified for the amount of β-actin expression compared to that of the controls for variation of samples (data not shown). The standard deviation of each sample was calculated from three independent experiments in duplicate.

### 4.7. The Detection of Copy Number of the EBV Genome

To detect released EBV virions, NA cells (1 × 10^6^ cells/well) were incubated with TPA (40 ng/mL) and SB (3 mM) for 48 h after pretreatment with kaempferol (0, 10, 20 and 50 μM) for 1 h. The supernatants were filtered through a 0.45 mM filter and incubated with 2 μL DNase I and 10× DNase I buffer (New England Biolabs, Ipswich, MA, USA). Each sample was then treated with 0.1 mg/mL proteinase K with a ratio of 1:1 [*v*/*v*] at 50 °C for 1 h. The reaction was stopped by heating the sample to incubate at 75 °C for 20 min. The sample was detected for the genome of the EBV DNA polymerase (BALF5) by real-time PCR analysis (sense: 5′-CGGAGTTGTTATCAAAGAGGC-3′; antisense: 5′-CGAGAAAGACGGAGATGGC-3′) [65].

### 4.8. Quantitative PCR Analysis

Quantitative PCR (qPCR) was performed according to the manufacturer’s instructions (Kapa Biosystems Fast qPCR kit). The qPCR conditions were: 5 s denaturation at 95 °C, 20 s annealing at 60 °C and 2 s extension at 72 °C for 45 cycles with specific primers of EBV genes (Table 1). The specificity of the PCR reaction was controlled by melting curve analysis (65–95 °C, 0.1 °C/s) in a LightCycler 480 (Roche Applied Science, Penzberg, Germany). Means and standard deviations were derived from three independent trials.

### 4.9. Immunofluorescence Analysis

Immunofluorescence analysis was performed to determine the population of NPC cells harboring lytic EBV [25]. Briefly, after treatment, cells were fixed with a 2% formaldehyde solution for 10 min and then permeabilized with 0.4% Triton X-100 in PBS for 5 min. The fixed cells were then blocked with 4% FCS-PBS for 30 min and treated with an anti-EAD antibody (1:10) for 1 h at room temperature. The secondary antibody, rhodamine-conjugated goat antimouse IgG (1:100), was added and incubated for 1 h. The cells were washed with 4% FCS-PBS, and 4′,6-diamidino-2-phenylindole (DAPI; 1:10,000) to stain the cell nuclei (Sigma-Aldrich). The results were observed using a fluorescence microscope (Olympus).

## Figures and Tables

**Table 1:** List of primers used for amplification qRT-PCR.

Target Gene		Oligonucleotide Sequence (5′→3′)
Zta	Forward	5′-GAGTCAACATCCAGGCTTGG-3′
	Reverse	5′-GCAGCACTACCGTGAGGTG-3′
Rta	Forward	5′AGAGACCAGAGAGCCCAGC-3′
	Reverse	5′-ACACAAACAGACGCAGATGAG-3′
EAD	Forward	5′-AGAGACACCCTCGCCTGC-3′
	Reverse	5′-GCTTCTGCTTCTGCCTGG-3′
DNase	Forward	5′-AGAAGGCCGTCACAATGTTC-3′
	Reverse	5′-TCCACGGCACAACTACTTCT-3′
gp350	Forward	5′-TTTCTGTGCCGTTGTCCC-3′
	Reverse	5′-CCCCACTGTATCCACCGC-3′
EBNA1	Forward	5′-GACAAAGCCCGCTCCTACCT-3′
	Reverse	5′-AGCCCCTTCCACCATAGGTG-3′
LMP1	Forward	5′-GGGTCGTCATCATCTCCACC-3′
	Reverse	5′-CCACACCTTCCTACGCTGC-3′
GAPDH	Forward	5′-CCTGCCAAATATTGATGACATCAAG-3′
	Reverse	5′-ACCCTGTTGCTGTAGCCAAA-3′

## Data Availability

Not applicable.

## Supplementary Materials

The following supporting information can be downloaded at: https://www.mdpi.com/article/10.3390/molecules27238158/s1. Figure S1: Kaempferol treatment inhibits EBV reactivation in Burkitt’s lymphoma cell line. Figure S2: The original films of Figure 2a in this manuscript. Figure S3: The original films of Figure 2b in this manuscript. Figure S4: The original films of Figure 7b in this manuscript.
