# Supplementary material for: The Dietary Flavonol Kaempferol Inhibits Epstein–Barr Virus Reactivation in Nasopharyngeal Carcinoma Cells
Source: Molecules. 2022 Nov 23;27(23):8158. doi: 10.3390/molecules27238158 (PMC9736733; doi:10.3390/molecules27238158)
Supplement: Supplementary file 1 [file molecules-27-08158-s001.zip › molecules-2010458-supplementary.pdf]

Supplementary Results

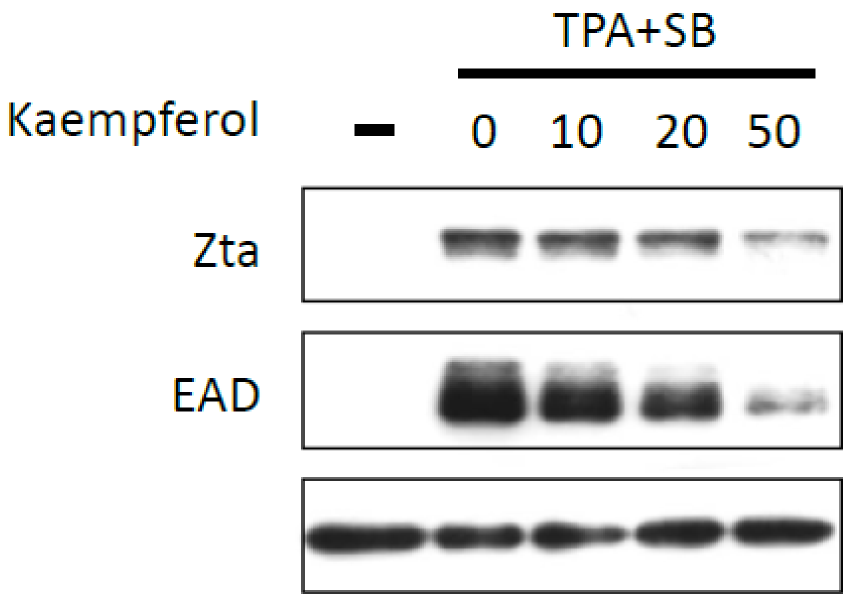

Supplementary Figure S1. Kaempferol treatment inhibits EBV reactivation in Burkitt's lymphoma cell line.

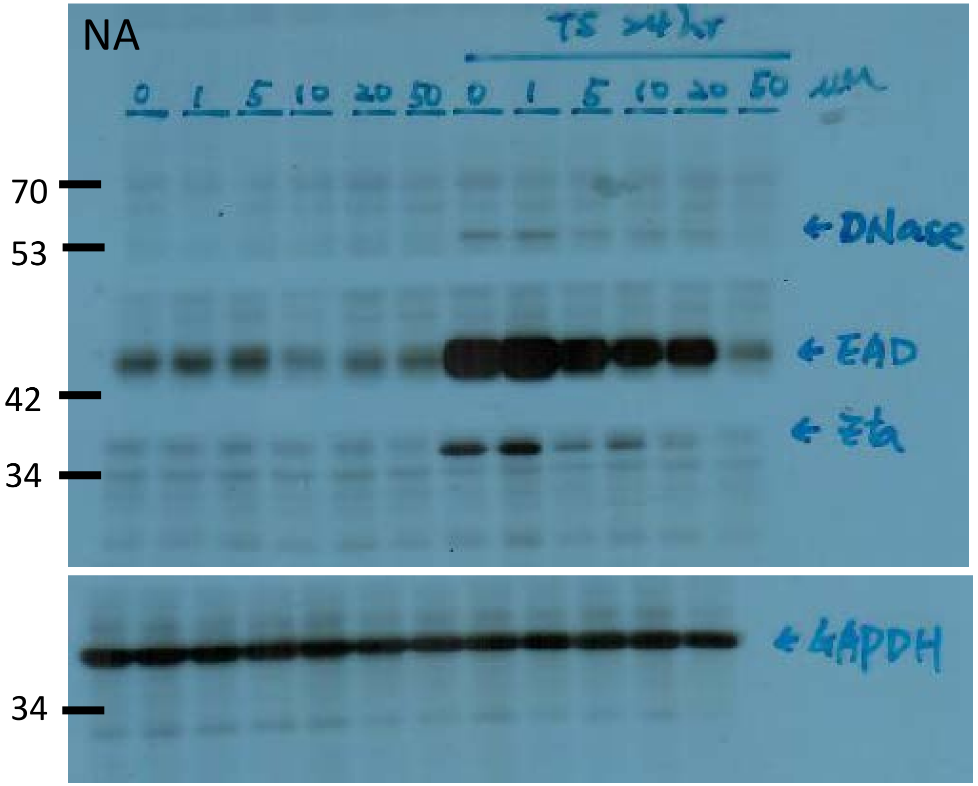

Supplementary Figure S2. The original films of Figure 2a in this manuscript.

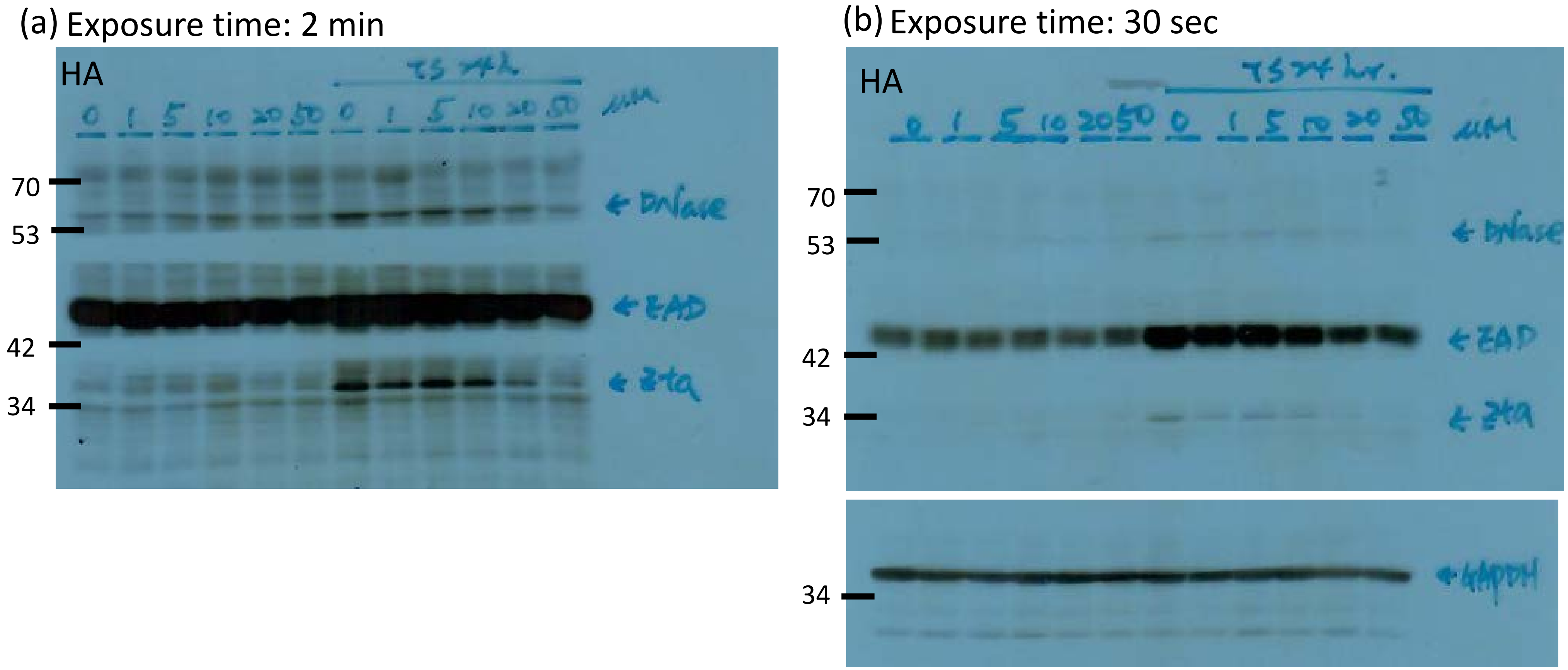

Supplementary Figure S3. The original films of Figure 2b in this manuscript.

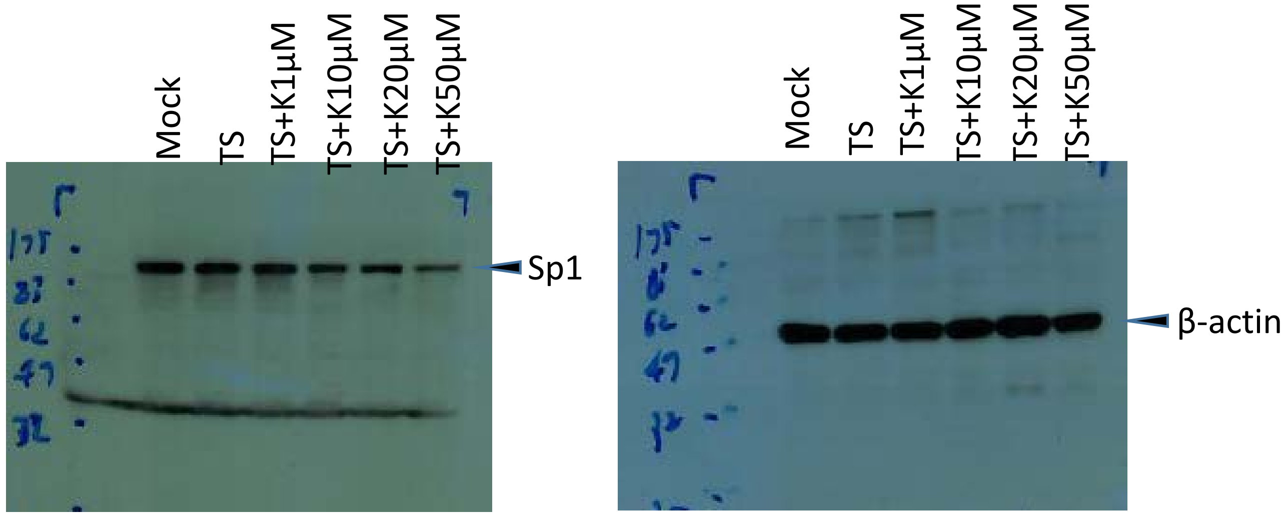

Supplementary Figure S4. The original films of Figure 7b in this manuscript.
